# Endothelial nitric oxide synthase is regulated by ERK phosphorylation at Ser^602^

**DOI:** 10.1042/BSR20140015

**Published:** 2014-09-17

**Authors:** John C. Salerno, Dipak K. Ghosh, Raj Razdan, Katy A. Helms, Christopher C. Brown, Jonathan L. McMurry, Emily A. Rye, Carol A. Chrestensen

**Affiliations:** *Department of Biology, Kennesaw State University, Kennesaw, GA 30144, U.S.A.; †Department of Chemistry and Biochemistry, Kennesaw State University, Kennesaw, GA 30144, U.S.A.

**Keywords:** eNOS, ERK, fluorescence lifetime, MAPK, nitric oxide synthase, phosphorylation, AI, autoinhibitory, Akt, PKB (protein kinase B), BAEC, bovine aortic endothelial cell, BH_4_, tetrahydrobiopterin, BLI, biolayer interferometry, CaM, calmodulin, eNOS, endothelial nitric oxide synthase, ERK, extracellular-signal-regulated kinase, iNOS, inducible nitric oxide synthase, MAPK, mitogen-activated protein kinase, nNOS, neuronal nitric oxide synthase, NOS, nitric oxide synthase, PKA, protein kinase A, PKC, protein kinase C, SPR, surface plasmon resonance

## Abstract

eNOS (endothelial nitric oxide synthase) contains a MAPK (mitogen-activated protein kinase)-binding site associated with a major eNOS control element. Purified ERK (extracellular-signal-regulated kinase) phosphorylates eNOS with a stoichiometry of 2–3 phosphates per eNOS monomer. Phosphorylation decreases NO synthesis and cytochrome *c* reductase activity. Three sites of phosphorylation were detected by MS. All sites matched the SP and TP MAPK (mitogen-activated protein kinase) phosphorylation motif. Ser^602^ lies at the N-terminal edge of the 42-residue eNOS AI (autoinhibitory) element. The pentabasic MAPK-binding site lies at the opposite end of the AI, and other critical regulatory features are between them. Thr^46^ and Ser^58^ are located in a flexible region associated with the N terminus of the oxygenase domain. In contrast with PKC (protein kinase C), phosphorylation by ERK did not significantly interfere with CaM (calmodulin) binding as analysed by optical biosensing. Instead, ERK phosphorylation favours a state in which FMN and FAD are in close association and prevents conformational changes that expose reduced FMN to acceptors. The close associations between control sites in a few regions of the molecule suggest that control of signal generation is modulated by multiple inputs interacting directly on the surface of eNOS via overlapping binding domains and tightly grouped targets.

## INTRODUCTION

Signalling networks include elaborate feedback and feed-forward mechanisms and are responsible for homoeostasis over multiple levels of organization. The familiar picture of signal transduction cascades in which amplification is obtained by sequential phosphorylation/activation has been augmented by the discovery of multi-component signalling complexes that may include receptors, kinases, scaffolds and adaptors, and non-kinase signal generators [1–4].

**Figure 1:**
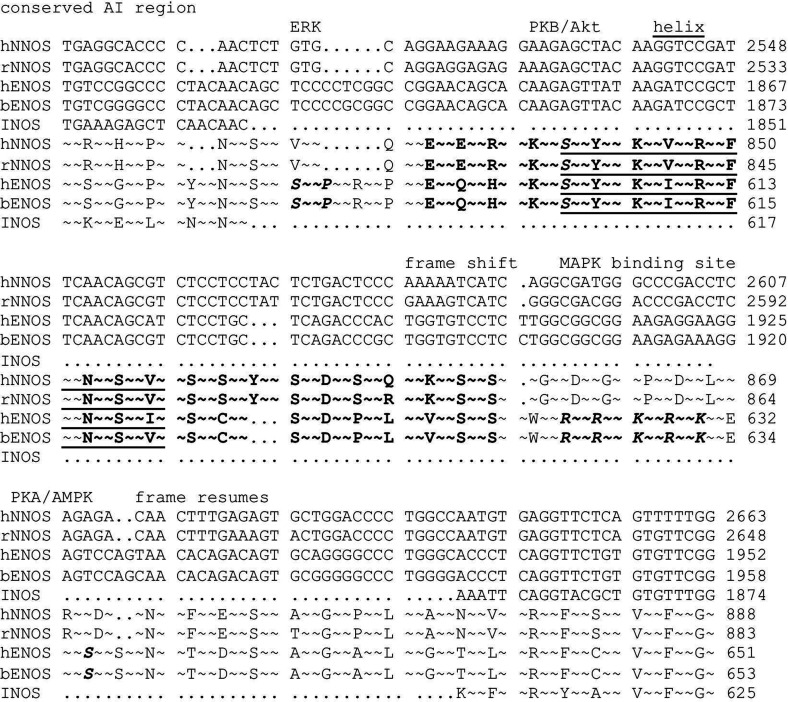
Sequence alignment of NOS mammalian isoforms in the AI element region Note conserved region (bold) and frame shifts causing divergence of the variable region in eNOS and nNOS. Helix residues are underlined. Phosphorylation sites for ERK, Akt and PKA/AMPK (adenosine monophosphate-activated protein kinase) and the binding site for MAPKs (ERK, p38) are marked with bold italics. Human sequences are denoted by h, rat sequences by r and bovine sequences by b. All DNA sequences numbered from the first base of the start codon in the most commonly studied splice form. Accession numbers for DNA: hnNOS, D16408; rnNOS, X59949; heNOS, M93718; beNOS, M99057; hiNOS, L24553. Protein accession numbers: hnNOS, P29475; rnNOS, P29476; heNOS, P29474; beNOS, P29473; hiNOS, P35228.

eNOS (endothelial nitric oxide synthase) is a signal generator in the regulation of vascular and airway tone, insulin secretion, angiogenesis and cardiac function [5–10]. Primary control is through Ca/CaM (calmodulin) activation [11], but numerous other inputs have been described including inhibitory and activating phosphorylation by specific kinases, protein-protein interactions and cell trafficking. [12–15] Examples of activating kinases include PKA (protein kinase A), which phosphorylates Ser^1179^ and Ser^635^, and Akt, which phosphorylates Ser^1179^ and Ser^617^ [15–18] (The sequence numbering of human and bovine eNOSs in the reductase regions differ by 2 amino acids; bovine Thr^497^ corresponds to human Thr^495^, bovine Ser^617^ and Ser^635^ to human Ser^615^ and Ser^633^ and bovine Ser^1179^ to human Ser^1177^; see also Figure 1). Ser^1177^ is located in the C-terminal tail, which acts to restrict the rate of haem reduction by the flavin containing domains [19–22]. Ser^635^ and Ser^617^ are located in a large AI (autoinhibitory) insertion in the FMN-binding domain, which is displaced by CaM binding during activation [23]. PKC (protein kinase C) inhibits eNOS by phosphorylating Thr^495^ [24,25], adjacent to the CaM-binding site, which interferes with CaM-mediated activation [26,27]. Other phosphorylation sites associated with the oxygenase domain are less well studied.

MAPKs (mitogen-activated protein kinases), including ERK (extracellular-signal-regulated kinase), p38 and JNK (c-Jun N-terminal kinase) are important signalling nodes in pathways that control metabolism, growth and expression [28–32]. ERK and p38 both function in signalling pathways [33,34] that involve eNOS and good evidence for direct phosphorylation of eNOS by ERK1/2 in BAECs (bovine aortic endothelial cells) has been obtained [35]. Contradictory reports of the site of ERK phosphorylation have appeared [36,37]. The uncertainty is the result of pathway-dependent phosphorylation events in which ERK drives the activation of other kinases, since some of the proposed sites do not match the target motifs of MAPKs, and may also reflect cross-talk in which phosphorylation at one position exposes other positions to phosphorylation.

We recently identified a pentabasic-binding site for MAPKs in the eNOS AI element [38]. Here we report the sites of ERK phosphorylation, the effects on activity, and the mechanism by which phosphorylation regulates NO synthesis. The phosphorylation sites are distinct from the D-domain-type binding site we reported previously. The uncertainties introduced by pathway-dependent phosphorylation events have been removed by working with purified components, but additional sites might be exposed by covalent modification or protein–protein interactions in cells.

## EXPERIMENTAL

Expression and purification of eNOS was carried out as previously described [37,39,40]. Prosthetic group content was measured spectrophotometrically [40]. NO synthase activity was measured using haemoglobin capture and Griess assay, and reductase activity was measured using cytochrome *c* reduction [41,42]. Affinity purified, single-band ERK2 from an *Escherichia coli* expression system was purchased from SignalChem.

ERK kinase reactions were performed in 20 mM Hepes (pH 7.4), 1 mM DTT, 10 mM MgCl_2_, 1 mM ATP, 10% (v/v) glycerol and when using the high concentration eNOS, 0.75 mM EGTA. eNOS was used at various concentrations (45–1.6 μM for fluorescence and eNOS/cytochrome *c* experiments, respectively). Reactions were done plus or minus ERK (0.12–0.01 μM, depending on eNOS concentration) at room temperature, ~22°C (see figure legends) and were ‘stopped’ by putting on ice until the activity was tested; within 5 h. 0.315 nM of phosphorylated and unphosphorylated eNOS were sent for MS analysis. MS of trypsin digested enzyme was carried out at the Emory University proteomics facility with duplicate phosphorylated and unphosphorylated samples.

Western blot analysis was done with a Li-Cor Odyssey. The control eNOS antibody was from Invitrogen (clone 9D10). The eNOS pS602 antibody is a peptide antibody. The phospho-peptide, used to immunize rabbits, and the resulting antibodies were affinity purified by NeoBioLab.

BAECs were purchased from Lonza, grown in the recommended media and harvested at confluence using lysis buffer (50 mM Tris, 150 mM NaCl, 1.5 mM MgCl_2_, 1% ipegal, 0.5% (v/v) Triton X-100, 2 mM EDTA, HALT protease inhibitor cocktail (Thermo) and 1 μM microcystin LR and 200 μM Na_3_VO_4_). Approximately 25% of the lysate from a 10 cm plate was treated with or without 400 units of λ phosphatase in a 50 μl reaction for the indicated times, 20 μl was loaded on the gel.

Time-resolved intensity decays were recorded using a PTI TCSPC (time-correlated single-photon counting) fluorescence lifetime spectrometer as described [43]. FMN was excited at 473 nm using a pulsed laser diode with 20 MHz repetition rate; experiments with 378 nm excitation produced similar results. The decay of fluorescence can be represented as the sum of individual exponential decays:

(1) $$I\left(t\right)=\sum_{ii-1}^{n}\exp\left(-\frac{t}{\tau_{i}}\right)$$

where the *τ_i_* are the decay times and *α_i_* are the amplitudes of the *i*th component. The fractional contribution of the *i*th component in the steady state is:

(2) $$f_{i}=\frac{\alpha_{i}\tau_{i}}{\sum {}_{j}\alpha_{j}\tau_{j}}$$

Individual values of *α_i_* and *τ_i_* were determined from simulation with PTI's Felix GX software with PowerFit 10 simulation module, using deconvolution of an instrument response function obtained from scattering and nonlinear least squares fitting to multiple exponentials. The quality of these fits were characterized by φ^2^. Additional information is available in [44].

BLI (biolayer interferometry) experiments were conducted essentially as described in [27]. Briefly, biotinylated CaM was immobilized on streptavidin sensors. After establishing a baseline in binding buffer (10 mM HEPES, pH 7.4, 100 mM NaCl, 10% glycerol, 10 μM CaCl_2_ and 0.05% surfactant P-20), sensors were moved to binding the buffer containing eNOS for 180 s. Sensors were then moved to the buffer only to monitor dissociation for 180 s. Non-specific binding was measured by immersing sensors without CaM in analyte and was negligible in all cases. All biosensing experiments were performed at 25°C.

SPR (surface plasmon resonance) experiments were conducted on a Biacore X100 instrument using a biotin CAPture chip. Biotinylated CaM (~150 RU in all cases) was immobilized prior to single-cycle kinetics performed in the binding buffer.

## RESULTS

Figure 1 shows the alignment of the DNA and amino acid sequences of mammalian NOS (nitric oxide synthase) isoforms in a region corresponding to an α–β turn within the Rossmann-fold FMN-binding domain. The signal generators eNOS and nNOS (neuronal nitric oxide synthase) differ from the cytokine induced isoform iNOS (inducible nitric oxide synthase) in that they have an extended insertion at this point that serves as an AI element displaced by CaM binding to a spatially adjacent site [23].

The N-terminal half of the AI, shown in bold, contains a conserved helical region (underlined residues) that locks down the FMN-binding domain through hydrogen bonds to both the FMN-binding domain and the NADPH-binding domain. This prevents conformational changes that are an obligatory part of the catalytic cycle. Ser^617^ and Ser^635^ in bovine eNOS (Ser^615^ and Ser^633^ in human eNOS) are phosphorylated by kinases [Akt (also known as PKB (protein kinase B)) and PKA] that activate the enzyme; Ser^1179^ in the inhibitory C-terminal extension is a target of the same kinases [15–18].

Recently we reported that MAPKs bind to a pentabasic sequence present in eNOS but not nNOS [38]. This site, shown in bold italics, confers 80 pM affinity for p38 and 160 nM for ERK. The location in this regulatory region suggests interactions between MAPKs and other modulators of eNOS activity.

The evolutionary origin of the MAPK-binding site is implied by the frame shift in the eNOS sequence in comparison to nNOS at the end of the conserved AI region [38]. As shown in Figure 1, a compensating shift restores the reading frame 27 bp later in eNOS, and the homology at the amino acid level immediately resumes. During the 27 bp frame shift, there is however no relationship between eNOS and nNOS at the amino acid level (some homology appears to persist at the DNA level). Divergence of eNOS from nNOS occurred during or before the development of amphibians, and translation of the nNOS sequence in Figure 1 with the appropriate frame shift introduces a basic motif. It seems plausible that these motifs arose by chance and were stabilized during evolution by protein–protein interactions.

**Figure 2:**
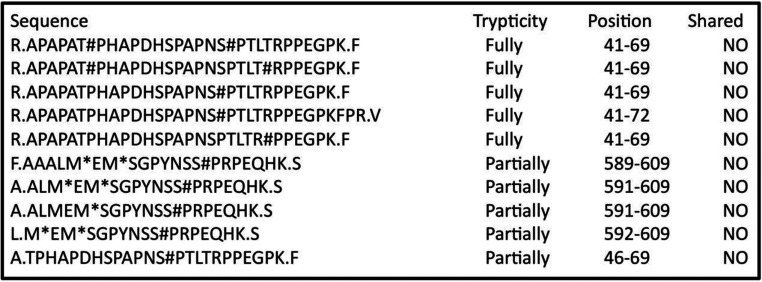
MS results for trypsin digest of ERK phosphorylated eNOS showing phosphorylated peptides Potential phosphorylation sites are indicated by # symbol. Because ERK phosphorylates TP and SP motifs, the results are uniquely consistent with Ser^602^, Thr^46^ and Ser^58^ phosphorylation, which can account for all the species observed. Asterisks indicate oxidized methionines. No peptides were shared with the unphosphorylated control.

Phosphorylation of eNOS by ERK was detected *in vitro* by measurement of ADP; the results showed that during a 1 h incubation, two or three ADP were formed per eNOS monomer, suggesting that multiple sites were phosphorylated. Figure 2 shows the results of MS of tryptic digests of unphosphorylated eNOS and eNOS phosphorylated for 15 min by ERK. Coverage was essentially complete; 35 phosphorylated tryptic peptides were obtained in addition to 3545 unphosphorylated peptides.

Ser^602^ and Thr^46^ were identified unambiguously as phosphorylation sites from the MS results alone, considering that only serine and threonine are potential targets. In addition, the data showed that either Ser^58^ or Thr^62^ was phosphorylated; because MAPKs phosphorylate at SP or TP sites, the third position was unambiguously identified as Ser^58^. No indication of phosphorylation at other sites was observed.

**Figure 3:**
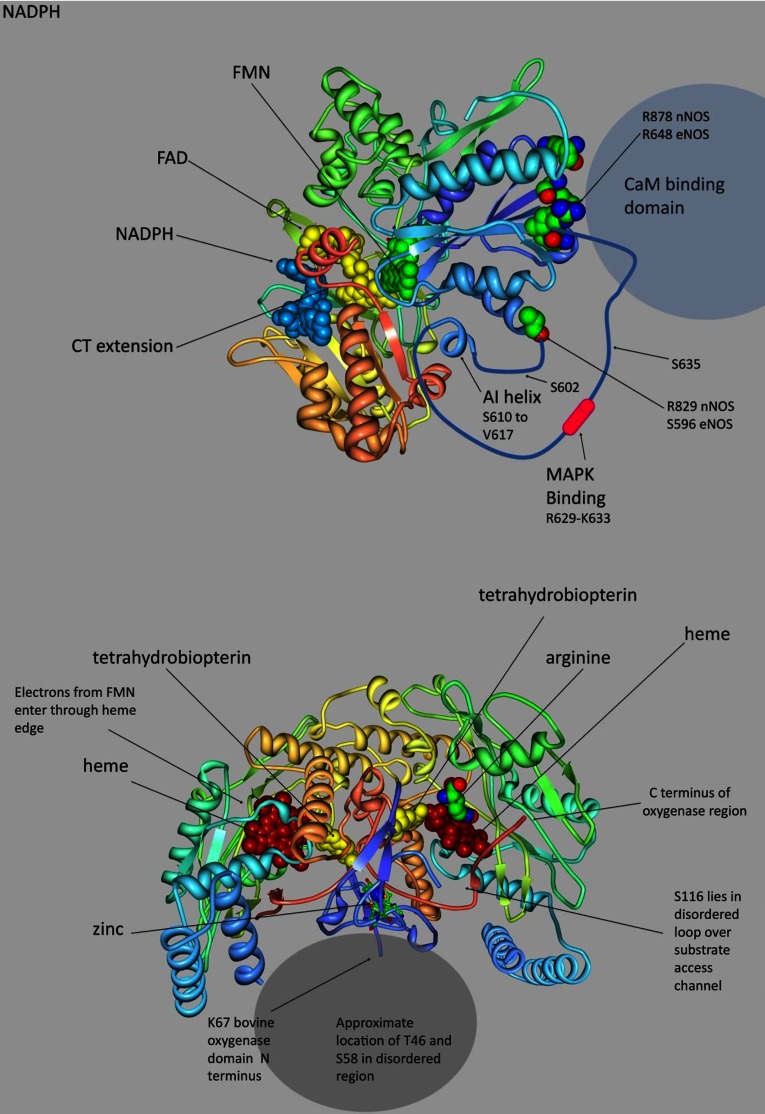
Location of phosphorylation sites in eNOS (**A**) Structural cartoon of NOS reductase domains based on the nNOS reductase crystal structure [45]. The FMN-binding domain is in blue, the FAD-binding domain green and the NADPH-binding domain is tan. Cofactors are shown in solid render, and the residues at the N-terminal edge of the FMN-binding domain and at the ends of the AI insertion (Arg^878^/Arg^648^ and Arg^829^/Ser^596^ for nNOS/eNOS) are marked by solid render. The approximate extent and position of the disordered regions of the AI are indicated by a drawn coil. The AI carried previously identified targets for Akt and PKA phosphorylation as well as a MAPK-binding site and a target for ERK phosphorylation (Ser^602^) that also matches the target motifs of other MAPKs. The C-terminal extension carries Ser^1179^, a target for Akt and PKA (see PDB 1TLL). (**B**) Structural cartoon of eNOS oxygenase dimer based on crystal structures [46,47]. The direction of the backbone is indicated by colour, with the N-terminal blue and the ribbon shading through green to tan at the C-terminal. Cofactors and substrate arginine are shown in solid render, with haem shown in red and BH_4_ in yellow. The location of two ERK targets in a disordered region at the bottom of the figure is indicated; the region is near the dimer interface. The C-terminal end of the domain is shown emerging from the face at right; this becomes the CaM-binding site, and after a short connector joins the FMN-binding domain shown in Figure 3(A). This domain must supply electrons to the haem at right by disengaging from the reductase complex and re-orienting (see PDB 4NSE and 3NOS).

Figure 3(A) shows the structure of the reductase portion of nNOS, corresponding roughly to the C-terminal half of the enzyme [45]. No corresponding eNOS crystal structure is available, but the two enzymes are highly homologous in this region and the conserved structures shown should be nearly identical. The ribbon diagram traces the path of the backbone through the FMN, FAD and NADPH-binding domains, with the cofactors shown in solid render. In this conformation, the FAD and FMN isoalloxazines are in van der Waals contact. At the opposite edge of the FMN-binding domain β sheet, the ends of the AI are exposed at the adjacent ends of an α-helix and a β-strand; the trace appears discontinuous because the chain is too flexible here to have a well-defined structure. The AI helix is visible as a disconnected feature, and is connected to the FMN domain β sheet by a long-disordered loop on the C-terminal side and a short-disordered loop on the N-terminal side. The long loop carries the MAPK-binding site close to the edge of the β-sheet and the adjacent CaM-binding site, accounting for the CaM–MAPK competition, which we previously reported [38]. Ser^602^ is located in the short-disordered loop directly adjacent to the α-helical region that precedes the AI. Ser^617^ is located in the AI helix, and Ser^635^ is in the long-disordered loop between the MAPK-binding site and the β-strand that follows the AI. (These residues correspond to Ser^600^, Ser^615^ and Ser^633^ in human eNOS.)

Figure 3(B) shows the structure of the eNOS oxygenase domain dimer [46,47]. The haem and BH_4_ (tetrahydrobiopterin) cofactors are shown in solid render along with the Zn atom that stabilizes the dimer. Thr^46^ and Ser^58^ are located in a disordered region adjacent to the loops bearing the cysteine residues that coordinate the Zn; the sites of myristoylation and palmitoylation are further towards the N-terminus. This region of the oxygenase domain surface is close to the point where the polypeptide chain leaves the oxygenase domain to form the CaM-binding site, and also reasonably close the site of haem reduction on the opposite monomer.

The results of experiments showing the effect of ERK-mediated phosphorylation on eNOS activity in NO synthesis and cytochrome *c* reduction are summarized in Table 1. These figures suggest inhibition of around 60%, but the results are slightly complicated by two factors. To obtain a high degree of phosphorylation, it is necessary to incubate eNOS with kinases at 22°C; during the incubation, control eNOS activity decreases slightly. If phosphorylated eNOS is less stable than unphosphorylated eNOS, we could overestimate inhibition slightly. A more serious problem is the incomplete phosphorylation of eNOS. Although we have several measures of the degree of phosphorylation (e.g., the results of fluorescence decay experiments), we cannot exclude the possibility that a minority population of unphosphorylated eNOS accounts for much of the residual activity.

**Table 1:** Effects of ERK catalysed phosphorylation of eNOS on activity and on the population of eNOS conformational states characterized by FMN fluorescence lifetimes The lifetime distributions in this table differ somewhat from those in the fits shown in Figure 5 because they are derived from decays of the CaM-activated enzyme to correspond to NO synthase activity. The distributions in Figure 5 show the effects of phosphorylation in the absence of CaM.

			Fluorescence lifetime (% state populations)		
	NO synthesis (nm^−1^ min^−1^ mg^−1^)	Cytochrome *c* reduction (min^−1^)	90 ps	0.9 ns	4.3 ns
eNOS	110 (10)	143 (20)	53.45	20.21	26.35
eNOS+ERK	42 (7.6)	55 (5)	85	–	15

We are confident that NO synthesis measured spectrophotometrically through the reaction of NO with oxyhaemoglobin is inhibited by at least 50%. Inhibition was confirmed in end point assays with the Griess reagent. Reduction of ferri-cytochrome *c* by eNOS was monitored at 418 and 550 nm to assess the effect of phosphorylation on electron transfer within the reductase unit. Phosphorylation also inhibits cytochrome *c* reduction by at least 50%. In both cases, it is possible that phosphorylation reduces the activity by more than 70%.

**Figure 4:**
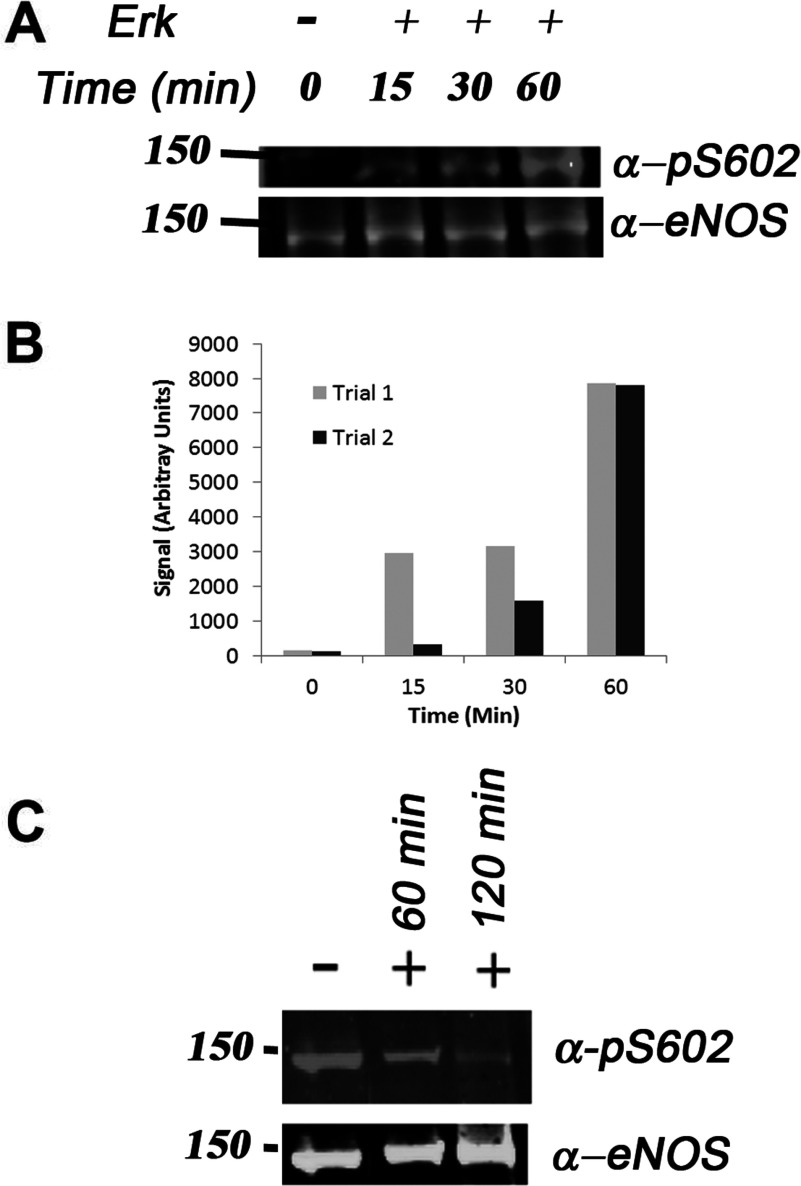
Time course of Ser^602^ phosphorylation and dephosphoryaltion (**A**) Western blots of purified eNOS after 0, 15, 30 and 60 min treatment with ERK showing the development of a band corresponding to phosphorylated Ser^602^ when probed with phospho-specific Ser^602^. Control probed with anti-eNOS shows that lanes are equally loaded and that eNOS is not significantly lost at times up to 1 h, but at longer times the signal from anti-eNOS is markedly reduced. (**B**) Plot of densitometry from two trials of time course. (**C**) Western blots of BAEC lysates treated with λ phosphatase and probed with anti-pS602 and anti-eNOS.

Figures 4(A) and 4(B) show the time course of eNOS phosphorylation using Western blotting with a phospho-specific pS602 antibody raised to a phosphorylated target peptide and depleted of unspecific components by treatment with unphosphoryalted peptide. After a lag of approximately 10 min, ERK phosphorylates eNOS with maximum intensity obtained at about 1 h. By 90 min, the control blot with anti-eNOS was significantly lower, and this was reflected in a weaker signal with anti-pS602.

Figure 4(C) shows Western blots of BAEC lysates probed with anti-pS602 and anti-eNOS after treatment with λ phosphatase. A strong band from eNOS phosphorylated at Ser^602^ is removed by the phosphatase over a period of 2 h, during which the anti-eNOS signal is essentially invariant. This demonstrates that Ser^602^ is significantly phosphorylated in endothelial cells, and that the anti-pS602 antibody does not bind to unphosphorylated eNOS.

**Figure 5:**
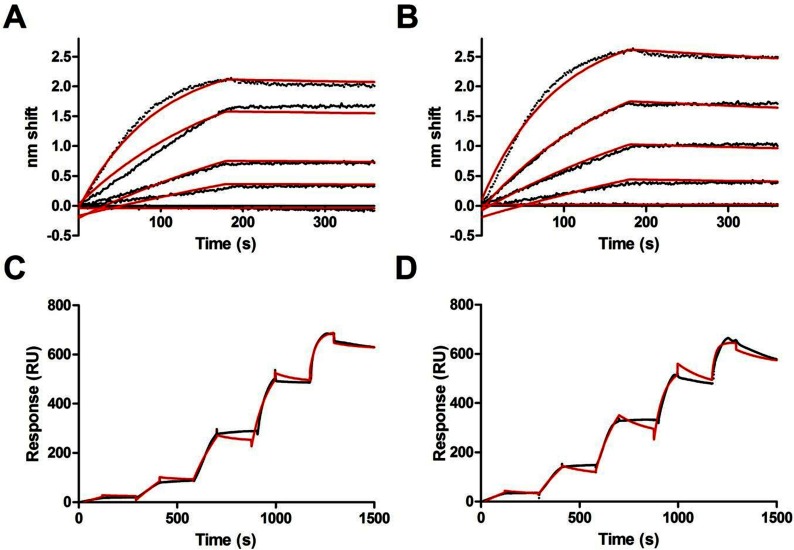
Optical biosensing analysis of CaM–eNOS binding (**A**) BLI sensorgram of CaM binding to 0, 10.9, 31.9, 43.7 and 87.5 nM unphosphorylated eNOS. Raw data are in black; fits to a single-state association-then-dissociation model are shown in grey. (**B**) Same as in (**A**), but for the same concentrations of phosphorylated eNOS. (**C**) SPR sensorgram of single-cycle kinetics of tethered CaM binding to 6.2, 18.5, 55.6, 167 and 500 nM analyte unphosphorylated eNOS. Reference-subtracted raw data are in shown in black along with fits to a two-state sequential model in red. (**D**) Same as in (**C**), but with phosphorylated eNOS.

We previously showed that CaM binding to unphosphorylated eNOS was diffusion limited and that PKC inhibition of eNOS via phosphorylation at Thr^497^ strongly inhibits CaM binding [27]. ERK phosphorylation of the target residues does not strongly affect CaM binding, suggesting that PKC and ERK inhibit eNOS via different mechanisms. Optical biosensing experiments demonstrate near-1 nM affinities regardless of phosphorylation state. Figures 5(A) and 5(B) show BLI traces with fits to global single-state models for CaM binding to unphosphorylated and phosphorylated eNOS, respectively. Rate constants are shown in Table 2. At the concentrations examined binding can be well approximated by a single-state first-order model with remarkably similar profiles regardless of phosphorylation. The difference in affinity observed is entirely due to a 3-fold higher k_off_, though this may not be physiologically relevant (see the Discussion section).

**Table 2:** Rate and equilibrium constants for CaM binding to unphosphorylated and ERK phosphorylated eNOS

	**−**ERK	+ERK
*BLI (single-state)*		
*k*_on_ (M^−1^ s^−1^)	1.2×10^5^	1.2×10^5^
*k*_off_ (s^−1^)	1.1×10^−4^	3.3×10^−4^
*K*_D_ (pM)	920	2700
*SPR (sequential model)*		
*k*_on1_ (M^−1^ s^−1^)	6.4×10^4^	1.1×10^5^
*k*_off1_ (s^−1^)	2.0×10^−3^	2.5×10^−3^
*k*_on2_ (s^−1^	8.4×10^−3^	3.7×10^−3^
*k*_off2_ (s^−1^)	1.9×10^−4^	1.1×10^−4^
*K*_D_ (pM)	720	650

Single-cycle kinetic analysis by SPR also revealed that CaM binding of unphosphorylated eNOS in Figure 5(C) is highly similar to phosphorylated (Figure 4D). CaM was immobilized prior to injection of the analyte eNOS. Fits to a sequential model (A+B↔AB↔AB*) generate *K*_D_s similar to those determined from BLI (Table 2), indicating that the secondary event is a minor component.

We recently showed that iNOS passes through a series of obligatory conformational states during its catalytic cycle, including an input state in which the FMN-binding domain is closely associated with the FAD and NADPH-binding domains, an output state in which the FMN-binding domain is associated with the haem-containing oxygenase domain, and a series of open conformations in which FMN is not closely coupled to other prosthetic groups [43]. These states can be resolved by their very different fluorescence lifetimes; eNOS and nNOS have similar conformational states. CaM activation of eNOS and nNOS results in increased levels of the output and open states relative to the closed input state.

**Figure 6:**
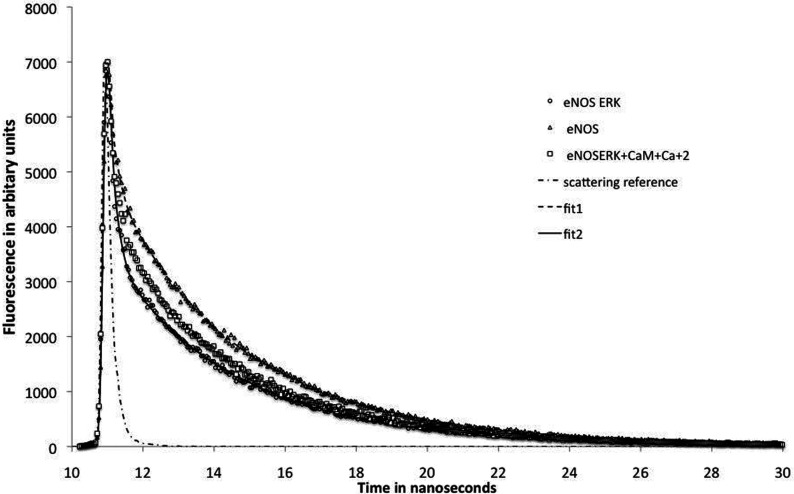
Fluorescence decays of eNOS holoenzyme FMN excited at 473 nm and detected at 525 nm The eNOS concentrations in the samples were 1 uM, and were from the same preparation. ERK phosphorylation favours conformational states with shorter lifetimes; CaM activation produces the opposite effect and as shown here partially reverses the effect of ERK phosphorylation. Fitting parameters were: unphosphorylated eNOS: 90 ps, 78%; 3.65 ns, 20%; 0.9 ns, 0.68%; 10.1 ns, 1.6%; *c*^2^=1.10. ERK phosphorylated eNOS: 80 ps, 86%; 3.83 ns, 12.4%; 1.1 ns 1.8%. All experiments were performed six times using at least two different preparations. The scattering reference was collected at 475 nm, and indicates the width of the exciting pulse.

Figure 6 shows fluorescence decays of unphosphorylated and ERK-phosphorylated eNOS in the presence and absence of CaM. ERK phosphorylation causes an increase in the input state, which has a lifetime of 90 ps because of close coupling between FMN and FAD, and a concomitant increase in the output state (0.9 ns lifetime) and the open states (4.3 ns average lifetime). This effect is in opposition to the effect of CaM binding, and accounts for the inhibition of NO synthesis and cytochrome *c* reduction by ERK-mediated phosphorylation.

The effects of ERK phosphorylation on eNOS activity and on the distribution of conformational states characterized by FMN fluorescence lifetimes are summarized in Table 1. The effects of ERK phosphorylation on the conformational manifold are opposite of the principal activator, CaM. Apparent inhibition of both NO synthesis and cytochrome *c* reduction is at least 60%, and may be greater because we are unable to phosphorylate eNOS completely without long incubations that damage the enzyme. Fluorescence data and information gained from phospho-specific antibody work suggest that the enzyme is at least 75% phosphorylated.

## DISCUSSION

The MAPKs, ERK and p38 are believed to participate in negative feedback signalling networks with eNOS, and good evidence indicates direct phosphorylation of eNOS by ERK in BAECs [35]. Based on indirect evidence obtained in intact cells, sites have been proposed for ERK phosphorylation including Ser^116^, Thr^497^ and Ser^635^ [36,37]. Of these, only Ser^116^ has the SP/TP motif associated with MAPK targets. Ser^116^ is located on the oxygenase domain, and the MAPK-binding site we recently identified [27] is located on the AI element of the FMN-binding domain. Phosphorylation of other residues (Thr^497^, Ser^635^ and human cognates) is the result of pathway dependent ERK-linked activation of other serine–threonine kinases.

Direct *in vitro* phosphorylation of purified eNOS with purified ERK confirmed that eNOS is a substrate for ERK, and further showed that phosphorylation did not require additional scaffolds or adaptors [35]. ERK phosphorylation inhibits both NO synthesis and cytochrome *c* reduction, indicating that phosphorylation interferes with electron transfer reactions mediated by FMN.

Three sites of phosphorylation on eNOS were identified; Ser^602^, at the N-terminal end of the AI, is spatially adjacent to the MAPK-binding site and the CaM-binding site, and is well positioned to interact with other control sites. A ‘lockdown’ of the FMN-binding domain is suggested by fluorescence results showing that phosphorylation pulls the enzyme's conformational distribution towards the input state. This accounts for the observed inhibition, and suggests that the negatively charged phosphate group stabilizes the input conformation. CaM appears to bind strongly to ERK phosphorylated eNOS, but is unable to effectively override phosphorylation imposed inhibition. Although the results of CaM activation and ERK phosphorylation on the fluorescence profile are fortuitously opposite ([49] and present communication), we point out that effects on the conformational equilibria are secondary to the changes in rates for conformational transitions. It is possible to inhibit the enzyme by locking it into any conformation, because the mechanism depends on conformational cycling [48].

Our previous report described in detail the complex kinetics of CaM–eNOS binding [27]. In the present study, ERK-phosphorylated eNOS demonstrated a similar CaM-binding profile; diffusion-limited association, very slow dissociation and picomolar affinity. A 3-fold slower dissociation rate constant in BLI accounts for the affinity difference between unphosphorylated and phosphorylated eNOS. The difference may be kinetically significant (95% confidence intervals for *k*_off_ do not overlap) but cannot account for differences in regulation of NOS activities. The difference in *k*_off_ is much less pronounced in SPR experiments and the affinities are nearly identical to the 650 pM previously reported [27]. In contrast, phosphorylation of Thr^497^ by PKC interferes with CaM binding [27]. The modes of ERK and PKC inhibition are thus entirely different: PKC prevents the binding of an activator, while ERK interrupts the catalytic cycle.

ERK phosphorylates eNOS at Thr^46^, Ser^58^ and Ser^602^; the first two of these residues are located in the N-terminal extension of the oxygenase domain. The location of Ser^602^ is ideal for the control of FMN-mediated electron transfer, and it lies close to the D-domain-type MAPK-binding site. The significance of Thr^46^ and Ser^58^ phosphorylation is unclear. The disordered N-terminal region is known to participate in protein–protein interactions, protein trafficking and myristoylation/palmitoylation. It is unclear that ERK bound to the AI pentabasic site can phosphorylate sites on the oxygenase domain.

We think it unlikely that MAPKs phosphorylate residues such as Thr^497^, Ser^617^, Ser^635^ and Ser^1179^. All these sites with the exception of Thr^497^ activate eNOS, and none of them has an SP or TP target motif [50,51]. When phosphorylation of these sites correlates with MAPK activation, it is likely that this occurs in a pathway-dependent manner. For inhibitory events such as Thr^497^ phosphorylation, this might represent parallel pathways of inhibition. For activating events, it probably represents feedback pathways involved in push–pull regulation.

We were surprised not to detect Ser^116^ phosphorylation. This residue is located on a flexible loop near the mouth of the substrate access channel of the oxygenase domain, is phosphorylated by SP-directed kinases *in vivo* and has been linked to ERK in plausible studies [37]. Several possibilities present themselves: phosphorylation could be mediated by other kinases, phosphorylation by MAPKs could require additional input (e.g., prior phosphorylation of other sites), or phosphorylation by MAPKs could require scaffolding components.

The tight spacing of control sites on the eNOS surface paints a picture of a system in which eNOS is at the junction of many signalling pathways and is an active node in the resulting network. A simple example of multiple inputs is Thr^497^ phosphorylation, which interferes with CaM binding and hence activation [24,27]. The sites associated with the AI are far more complex. Activating kinases such as PKA and Akt phosphorylate multiple targets in this region (12–18), which also includes the ERK/p38-binding site, the Ser^602^ ERK target and the AI helix, which locks the FMN-binding domain to the NADPH-binding domain. It is likely that phosphorylation of these sites affects the binding of other regulators and phosphorylation of other sites. The complexity of the system may allow eNOS to behave more like an integrated circuit with multiple inputs and a nuanced array of outputs than a simple relay.
